# Appearance of tuft cells during prostate cancer progression

**DOI:** 10.1038/s41388-023-02743-1

**Published:** 2023-06-29

**Authors:** Katarina Vlajic, Hannah Pennington Kluger, Wenjun Bie, Bradley J. Merrill, Larisa Nonn, Andre Kajdacsy-Balla, Angela L. Tyner

**Affiliations:** 1 Department of Biochemistry and Molecular Genetics, University of Illinois at Chicago, Chicago, IL 60607 USA; 2 The University of Illinois Cancer Center, Chicago, IL 60607 USA; 3 The Department of Pathology, at the University of Illinois at Chicago, Chicago, IL 60607 USA

**Keywords:** Cancer, Biomarkers

## Abstract

Tuft cells are chemosensory epithelial cells that increase in number following infection or injury to robustly activate the innate immune response to alleviate or promote disease. Recent studies of castration resistant prostate cancer and its subtype, neuroendocrine prostate cancer, revealed *Pou2f3+* populations in mouse models. The transcription factor *Pou2f3* is a master regulator of the tuft cell lineage. We show that tuft cells are upregulated early during prostate cancer development, and their numbers increase with progression. Cancer-associated tuft cells in the mouse prostate express DCLK1, COX1, COX2, while human tuft cells express COX1. Mouse and human tuft cells exhibit strong activation of signaling pathways including EGFR and SRC-family kinases. While DCLK1 is a mouse tuft cell marker, it is not present in human prostate tuft cells. Tuft cells that appear in mouse models of prostate cancer display genotype-specific tuft cell gene expression signatures. Using bioinformatic analysis tools and publicly available datasets, we characterized prostate tuft cells in aggressive disease and highlighted differences between tuft cell populations. Our findings indicate that tuft cells contribute to the prostate cancer microenvironment and may promote development of more advanced disease. Further research is needed to understand contributions of tuft cells to prostate cancer progression.

## Introduction

Tuft cells are solitary chemosensory cells found throughout epithelia of different organs (reviewed in [1]). Their numbers can increase dramatically upon infection, injury, or disease. Tuft cells express markers that differentiate them from surrounding epithelial cells, including POU2F3 (POU class 2 homeobox) a master transcription factor [2, 3], and TRPM5 (transient receptor potential isoform M5) [4]. While DCLK1 (Doublecortin-like kinase 1) is the most common marker for tuft cells, it is only expressed in murine tuft cells [5, 6]. Expression of taste receptors and the succinate receptor 1 (SUCNR1) on tuft cells enables them to detect environmental changes in metabolites [7, 8]. They are also characterized by expression of signal transduction genes characteristic for taste buds [1]. Activation of taste/chemosensory receptors on tuft cells transduces environmental signals to other effector systems. The most studied effector function is activation of type 2 immunity through secretion of interleukin (IL)-25 that activates type 2 innate lymphoid cells (ILC2s), leading to secretion of IL-13 and other cytokines [3, 9, 10]. Furthermore, tuft cells express enzymes required for bioactive lipid synthesis, best characterized for production of prostaglandins, leukotrienes [1].

Tuft cells may suppress or promote carcinogenesis [1]. A subpopulation of tuft cells with properties of tumor stem cells was identified in mouse intestinal tumors [5, 11]. Tuft cell numbers increase in pancreatic metaplasia and neoplasia stages, but gradually decrease as cancer progresses [12–15]. However, *Pou2f3* is required for pancreatic cancer metastasis [14]. Upregulation of POU2F3+ tuft cells is also detected in a subset of small cell lung cancers, characterized by the absence of neuroendocrine markers [16].

Prostate cancer is the second leading cause of death from cancers in men, and metastasis results in a decreased 5-year survival rate of 30% [17]. Accumulation of mutations and gene alterations in epithelial cells are the main drivers of prostate cancer development and progression. Activation of androgen receptor (AR) signaling, through both hormone-dependent and -independent mechanisms, promotes cancer progression. In addition, mutations in *PTEN*, *RB1*, *TP53* and upregulation of *MYCN*, also contribute to advanced prostate cancer [18, 19] (reviewed in [20]). These changes occur in castration-resistant prostate cancer (CRPC), which most commonly develops after the first line of treatment, androgen deprivation therapy. CRPC is characterized by either androgen-independent activation of AR, or development of AR-negative cancers like neuroendocrine prostate cancer (NEPC) [20]. Recently, *POU2F3* and several tuft cell markers, but not *TRPM5*, have been identified in prostate adenocarcinoma [21]. Furthermore, single cell (sc) RNA-seq analysis of mouse models of aggressive disease revealed the existence of different types of neuroendocrine populations, marked by expression of *Ascl1* and *Pou2f3* [19], or *Pou2f3*+ tuft cells [22].

Growing recognition of the importance of tuft cells in epithelial biology and cancer led us to explore the presence of tuft cells in the healthy prostate and prostate cancer, and their correlation with aggressive disease. Our studies indicate that tuft cells are present in prostate tumors in mice and men, and their numbers increase as cancer progresses. Examining contributions of tuft cells to signaling and the tumor microenvironment may further our understanding of prostate cancer progression and facilitate the development of therapeutics to treat advanced prostate cancer.

## Results

### Tuft-like cells are present in mouse models of prostate cancer

**Fig. 1:**
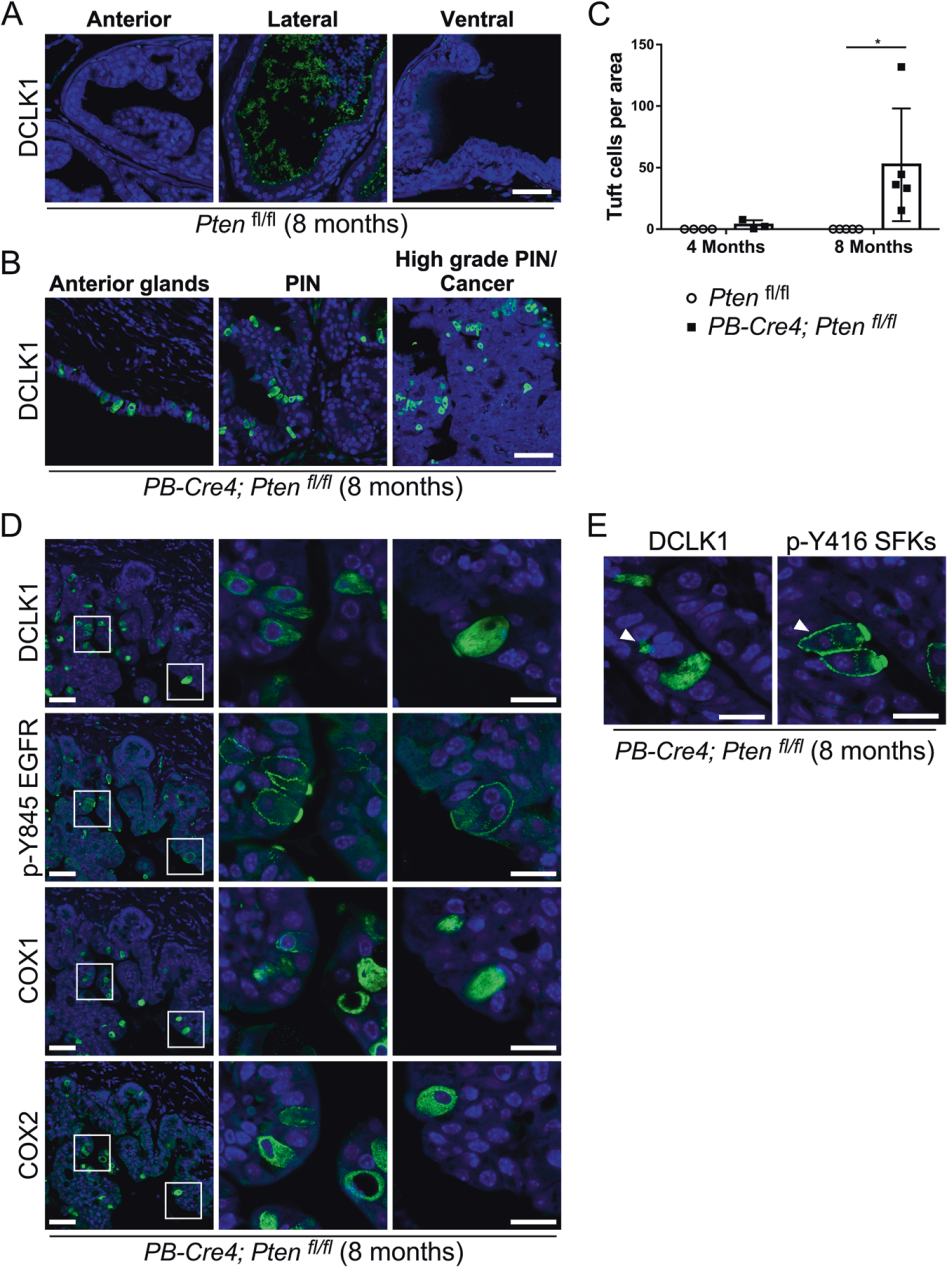
Tuft-like cells are upregulated in a mouse model of prostate cancer. Intact prostates were removed and all lobes were analyzed by immunofluorescence staining. **A** Tuft cells are not detected in prostates from healthy 8-month-old *Pten*^fl/fl^ (control) mice. Scale bar: 50 μm. **B** DCLK1+ tuft-like cells are found in prostate tissues from 8 month-old *PB-Cre4*;*Pten*^fl/fl^ mice with prostate specific disruption of *Pten*. Scale bar: 50 μm. **C** Quantification of DCLK1+ tuft cells in prostate tissue sections from 4 and 8-month-old mice. N (control, 4 months) = 4, N (control, 8 months) = 5, N (*PB-Cre4;Pten*^*fl*/fl^, 4 months) = 3, N (*PB-Cre4;Pten*^fl/fl^, 8 months) = 5; Error bars shown as ± SD. (*) *p*-value < 0.05. **D** Expression of DCLK1, COX1, and COX2, and activating phosphorylation of EGFR (p-Y845) is detected in the same cells in *Pten*-null prostates. Serial sections of prostate tissue from 8 month-old *PB-Cre4*;*Pten*^fl/fl^ mice are stained for tuft cell-related proteins. Scale bar: 50 μm for lower magnification, 20 μm for higher magnification. **E** Tuft cells in prostate exhibit strong activation of SRC family of kinases (SFKs). Prostate tissue from 8 month-old *PB-Cre4;Pten*^fl/fl^ mice stained DCLK1 and p-Y416 SRC (which cross-reacts with activating phosphorylation of all SFKs) shows localization of both proteins in the same cells in adjacent sections. Scale bar: 20 μm.

Tuft cells have not been characterized in the prostate. We compared normal mouse prostates to those with conditional disruption of *Pten* in the prostate (*PB-Cre4;Pten*^*fl/fl*^). *Pten*-null mice develop adenocarcinomas as early as 17-26 weeks of age, without the neuroendocrine phenotype [23]. Using DCLK1 as a marker for murine tuft cells, we stained sections of intact prostate with all lobes present. We did not detect any DCLK1+ tuft-like cells in prostates of 8-months old control mice lacking *Cre4* and expressing *Pten* (Fig. 1A). However, we observed DCLK1+ single cells only in the anterior lobes, in ductal structures, prostatic intraepithelial neoplasia (PIN), and in high-grade PIN and cancerous regions from 8-month old mice with disruption of *Pten* in the prostate (Fig. 1B). The number of DCLK1+ tuft cells increases with age, as cancer progresses in *Pten*-null prostates (Fig. 1C). Prostate cells expressing DCLK1 were also positive for other markers of tuft cells, including COX1 and COX2, active tyrosine phosphorylated EGFR (p-Y845) [24], and active SRC family kinases (SFKs) (p-Y416) [25] (Fig. 1D, E).

### Tuft cell marker genes cluster with the *Pou2f3*+ populations in mouse models of prostate cancer, revealing genotype-related differences

**Fig. 2:**
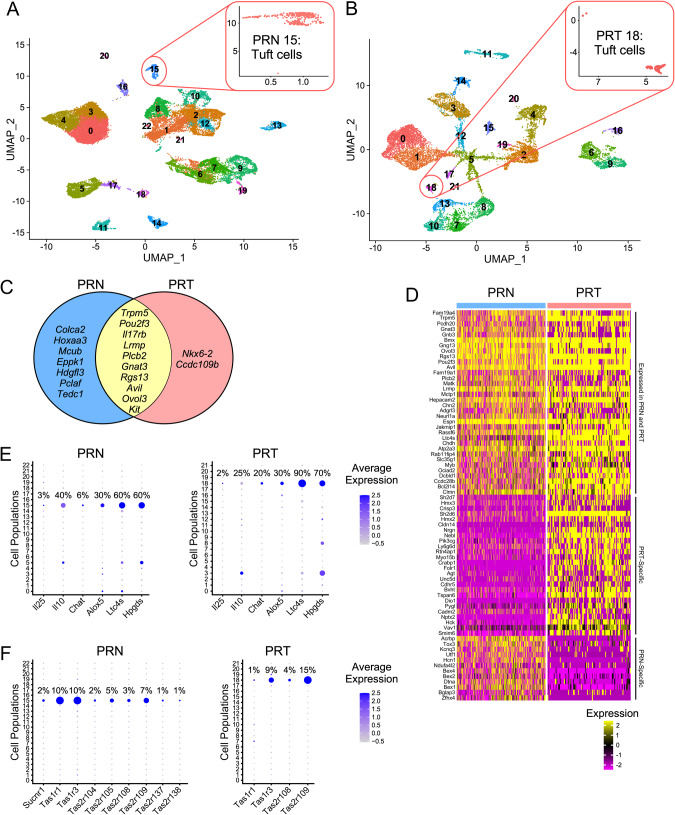
Tuft cell-related genes cluster into distinct populations in scRNA-seq data from mice with deletion of *Pten* and *Rb1*, and overexpression of *MYCN* (PRN), and deletion of *Pten*, *Rb1* and *Tp53* (PRT). **A** Re-clustering of PRN scRNA-seq data from Brady et al. [19] using 30 principal components for clustering resulted in 22 clusters, with cluster 15 expressing marker genes specific for tuft cells. Different cell types in the PRN dataset include: 0, 3, 4 – granulocytes; 1 – mitochondria; 2, 8, 10, 12, 20, 21, 22 – epithelial cells; 5, 17 – macrophages; 6, 7, 9 – fibroblasts; 11 – T cells; 13 – neuroendocrine cells; 14 – endothelial cells; 15 – tuft cells; 16 – seminal vesicle; 18 – B cells; and 19 – smooth muscle. **B** Re-clustering of PRT scRNA-seq data Chan et al. [22] using 30 principal components for clustering resulted in 21 clusters, with cluster 18 expressing marker genes specific for tuft cells. Different cell types in the PRT dataset include: 0, 1 – granulocytes; 2, 4, 17, 20, 21 – epithelial cells; 3, 12 – macrophages; 5 – N/A; 6, 9 – fibroblasts/Vim + ; 7, 8, 10, 13 – neuroendocrine cells; 11 – T cells; 14 – B cells; 15 – endothelial cells; 16 – smooth muscle/Vim + ; 18 – tuft cells; and 19 – epithelial cells/Tff3 + . Expanded UMAP for populations 15 and 18 are shown the upper right panels in **A** and **B**. **C** Genes expressed only in PRN mice (not found in PRT dataset, blue), only in PRT (not found in PRN dataset, light red), and specific markers for both datasets that have been described elsewhere (yellow) are shown in a Venn diagram. **D** Heatmap of tuft cell marker genes, including genes expressed in both datasets or specific for PRN or PRT tuft cell populations. The threshold is set as average logFC>0.6, p (adjusted) <10^-100^ and ratio (pct1/pct2) > 5. **E** Dotplot of genes involved in effector functions of tuft cells, with the percentage of populations that express specific genes indicates *Il25* expression in both genotypes is low. Prostate tuft cells specifically express the anti-inflammatory *Il10*, which has higher average expression in PRN mice. PRT tuft cells have higher percentage of cells expressing *Chat*. Genes for bioactive lipid synthesis are high in both PRN and PRT mice. **F** Dotplot of sensing receptor expression, including *Sucnr1* and taste receptors, with percentage of population that express them. PRT mice do not express *Sucnr1*.

Studies of mouse models for aggressive CRPC and NEPC demonstrated the presence of cell populations positive for *Pou2f3*+ [19, 22], a master regulator of tuft cell differentiation [2, 3]. We used scRNA-seq data from a mouse model with *Pten* and *Rb1* deletion and ectopic expression of *MYCN* (PRN) [19], and from a model with *Pten*, *Rb1* and *Tp53* deletion in the prostate (PRT) [22]. Analysis of scRNA-seq data from PRN and PRT mice revealed tuft cell populations in both genotypes (Fig. 2A, B; Table S2). Cell populations 15 (PRN) and 18 (PRT) express the tuft cell master regulator *Pou2f3*, as well as tuft cell specific markers and genes specific for signal transduction (Fig. 2C, D; S1A, B; Table S2). However, we also discovered unique genes for each population, as summarized in Fig. 2C, D.

To explore differences in gene expression between PRN and PRT tuft cells, we compared tuft cell gene expression in these two genotypes with known markers of different tuft cell types identified in lung and small intestine (SI) [1, 26, 27]. Tuft cells that develop in PRN prostates exhibit a type-1 (neuronal, less mature) tuft cell gene signature, while gene expression in PRT tuft cells resembles more type-2 (immune, more mature) tuft cells (Table 1, S2). Furthermore, the analysis of Tp53-regulated genes [28] indicates several tuft cell genes may be regulated by p53 in the PRN model (Table 1, S2). Similarly, analysis of NMYC-regulated genes [29] shows higher expression of NMYC-targeted genes in the PRN group than in the PRT group (Table 1, S2).

**Table 1:** Differentially regulated genes in mouse PRN and PRT tuft cells.

Transcriptionally regulated genes				Tuft cell type-specific genes							
PRN-specific		PRT-specific		PRN-specific				PRT-specific			
Tp53 genes	NMYC genes	Tp53 genes	NMYC genes	Lung		Small intestine		Lung		Small intestine	
				Type-1	Type-2	Type-1	Type-2	Type-1	Type-2	Type-1	Type-2
Cdh13	Map2	Rtn4ip1		Fras1				Ano7	Hmx2		Nrgn
Nfib	Prmt7	Ccdc109b		Hecw1				Pik3cg	Nrgn		Folr1
Tcf4		Col9a3		Il10				Ly6g6d	Sh2d6		Agt
Coro2b				Kcnq3				Prss53	Ackr4		Tspan6
Prom2				Coro2b				Tspan6	Col9a3		Hck
Bmp7				Zfhx2				Pygl	Strip2		
Pcbp4				Six1				Spire2	Bc016579		
Eppk1				Cdh13					Hck		
Tex9				Sptbn2					Vav1		
				Tcf4							

Tuft cell-specific genes identified in PRN and PRT genotypes were compared and genes that are regulated by Tp53 or NMYC were identified (left columns) to determine their possible contributions to tuft cell gene expression. PRN-specific tuft cells express more NMYC- and p53-regulated genes than PRT tuft cells. Tuft cell-specific genes identified for PRN and PRT genotypes were compared with genes characteristic for different tuft cell types: type-1 or neuronal and early tuft cells, and type-2 or more mature tuft cells with an immune related signature (right columns). We used type-1 and type-2 tuft cell marker genes previously identified in the small intestine and in lung to classify gene expression as type-1 or type-2. Even though they share many genes, PRN tuft cells express more type-1, while PRT tuft cells express more type-2 related signature genes. The threshold was set to show genes with *p* (adjusted) <100^-100^ (Table S2).

Exploring effector functions of PRN and PRT tuft cells, we identified small subpopulations of tuft cells that specifically express *Il25* and *Chat* (choline acetyltransferase), the gene required for acetylcholine (ACh) production [30] (Fig. 2E). As characterized in other tissues, prostate tuft cells express enzymes for bioactive lipid synthesis (*Alox5, Ltc4s, Hpgds*). While both PRN and PRT tuft cell populations express *Il10*, it is expressed higher in PRN mice, as indicated by average expression levels (Fig. 2E). Both populations express the sensing receptors – *Tas1r1*, *Tas1r3*, *Tas2r108*, and *Tas2r109* (Fig. 2F). PRN tuft cells also express *Tas2r104*, *Tas2r105*, *Tas2r137*, *Tas2r138* and the succinate receptor *Sucnr1* (Fig. 2F). Although proteins used to identify tuft cells are expressed in both genotypes and are not exclusive to tuft cell clusters (Fig. S1C), they can be used in combination with other markers such as phosphorylated kinases (Fig. 1) to identify tuft cells in prostate cancer.

### Tuft cell genes are upregulated with disease severity and age

**Fig. 3:**
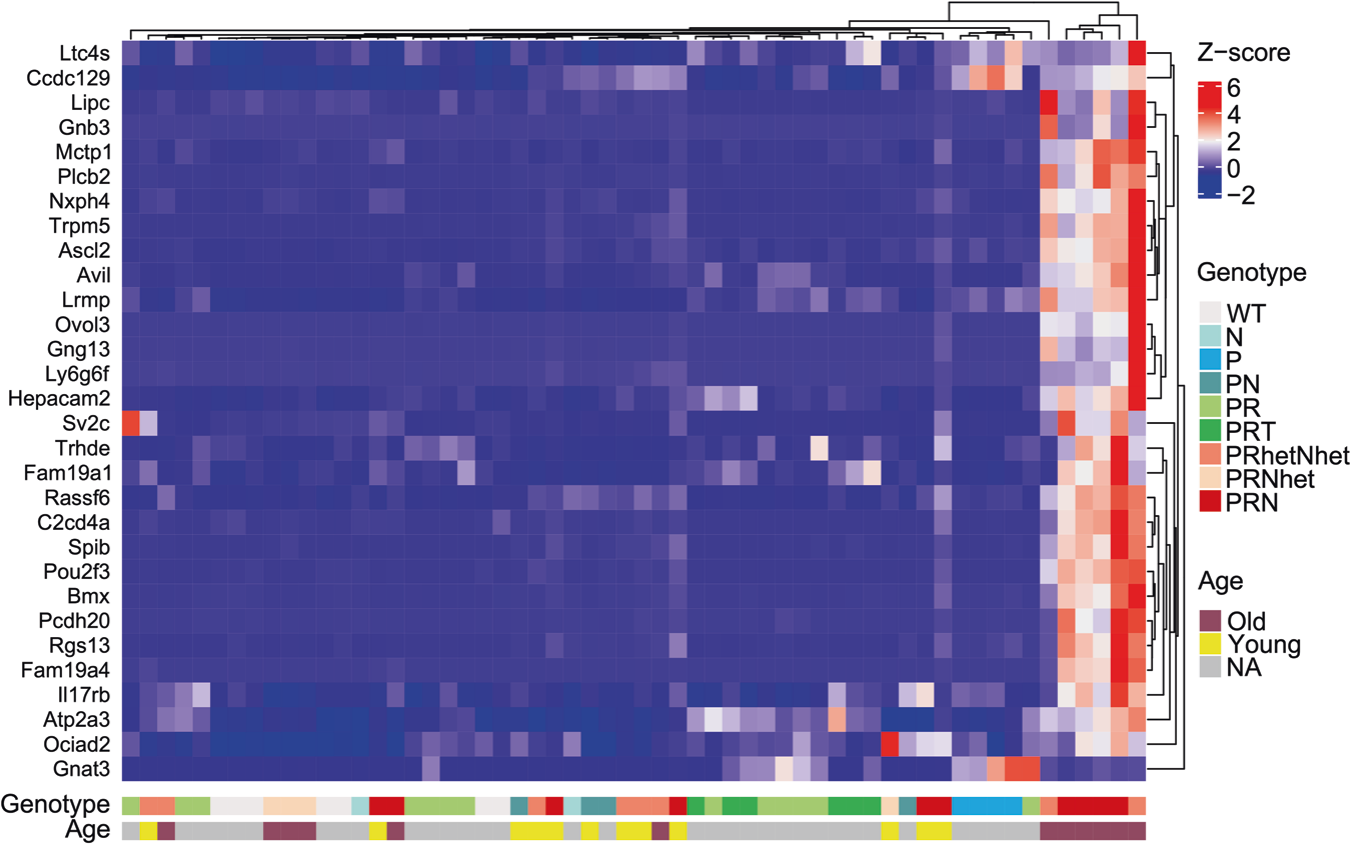
Tuft cell-related genes are markedly upregulated with age and expression of *MYCN* as the oncogenic driver. RNA-seq data from WT mice and mice with single, double, and triple genetic alterations: *Pten* deletion (P), *MYCN* overexpression (N), *Pten* deletion and *MYCN* overexpression (PN), *Pten* and *Rb1* deletion (PR), *Pten*, *Rb1* and *Tp53* deletion (PRT), and *Pten*, *Rb1* deletion and *MYCN* overexpression (PRhetNhet, PRNhet and PRN) show significant upregulation of tuft cell marker genes. Raw counts from studies GSE86532, GSE90891 and GSE158467 [18, 19, 31] were adjusted for batch effect and genes that are expressed in both PRN and PRT tuft cells (adjusted *p*-value < 10^-200^) were extracted from the batch-corrected dataset, scaled and the heatmap was generated using ComplexHeatmap function in R software. Clustering of gene expression revealed a group of aged PRN/PRhetNhet mice that have high tuft cell-related gene expression.

Brady and colleagues found that the *Pou2f3*+ prostate cell population increases with cancer progression [19], but Chan et al. showed that tuft cell numbers do not necessarily increase after loss of tumor suppressors and cancer progression [22]. Since we observed tuft cell expansion in *Pten*-null prostates, and in scRNA-seq data from both PRN and PRT mice [19, 22], we explored the correlation between tuft cell gene expression with disease aggressiveness. We adjusted for batch effects when analyzing the three RNA-seq datasets from mice with overlapping single, double and triple genetic alterations to determine contributions of different aggressive disease genotypes to tuft cell production. These datasets include 58 samples of all genotypes that mimic progression of disease from healthy prostate to neuroendocrine prostate cancer: GSE86532 with WT, N (*MYCN* overexpression) or PN (*Pten* deletion with *MYCN* overexpression) mice [18]; GSE90891 which includes WT, P (*Pten* deletion), PR (*Pten* and *Rb1* deletion) and PR with additional disruption of *Tp53* (PRT) [31]; and GSE158467, with data from PN and PRN (PRhetNhet, PRNhet and PRN) mice [19] (Table S3). Analysis of top tuft cell marker gene expression revealed significant upregulation in the combined dataset (Table S3). The heatmap of these genes shows clustering of a specific subgroup of aged PRN/PRhetNhet mice with particularly high levels of tuft cell gene expression (Fig. 3). Furthermore, we confirmed that there is significant upregulation of tuft cell marker genes in older mice (Table S3). This indicates that expression of tuft cell genes changes with progression of cancer. Tuft cell marker genes may be inherently upregulated in advanced prostate cancer, and further increase with progression with MYC as an oncogenic driver.

### Tuft-like cells are present in human prostate cancers

**Fig. 4:**
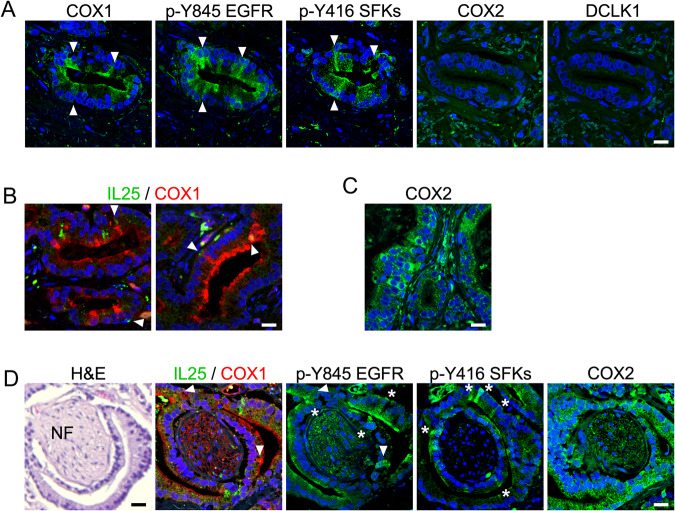
Tuft-like cells are present in human prostate cancer. **A** Tuft cells express COX1, active EGFR and active SFKs, but not COX2 and DCLK1 in human prostate cancer. Serial sections of tissues were stained for tuft cell-related proteins. Arrowheads show the same cell staining with three different antibodies. Scale bar: 20 μm. **B** IL-25 expression is high in samples that have tuft cells. Expression of IL-25 is found within or adjacent (secreted) to tuft cells and was visualized using double staining with anti-COX1 and anti-IL-25 antibodies. IL-25 is also high in adjacent cells in stroma. Scale bar: 20 μm. **C** COX2 does not label single tuft cells in prostate tissues, but other cancer areas. Staining of tissues with anti-COX2 does not show the same pattern of expression as COX1 and active kinases. Scale bar: 20 μm. **D** Serial sections, showing tuft-like cells around a nerve fiber (NF labeled in H&E), stained with anti-COX1, IL-25, and active EGFR (p-Y845) and active SFK (p-Y416) antibodies. The whole gland around a nerve visualized with COX2 staining, not showing tuft-like staining. Arrowheads show the same cell stained in adjacent sections. Asterisks show tuft-like morphology not present in adjacent sections. Scale bar: 20 μm.

To determine how our findings from mouse models translate to human disease, we stained prostate tissue isolated from patients diagnosed with CRPC for expression of tuft cell markers. We observed tuft-like cells in tissues isolated by transurethral resection of the prostate in 2 out of 4 patients. In human prostate cancers, tuft-like cells express COX1, active EGFR and active SFKs, but not DCLK1 and COX2 (Fig. 4A). IL-25 levels are high in these tissues, both adjacent to tuft cells and in cells in the stroma, presumably in immune cells. Staining of tissue sections with antibodies specific for COX1 and IL-25 indicates that some COX1+cells express IL-25 (Fig. 4B). We also observe IL-25 expression within glands without COX1 expression, which may represent secreted IL-25 from adjacent tuft cells that are not present in the tissue section. COX2 is present throughout tumors and adjacent stroma (Fig. 4C, D), but not in all glands with tuft-like cells (Fig. 4A) and does not have a tuft cell-like pattern of expression. COX1+ tuft-like cells in human prostate cancer appear concentrated around nerve fibers (NF) (Fig. 4D).

### DCLK1 is expressed in human prostate, but it is not a marker of tuft cells

**Fig. 5:**
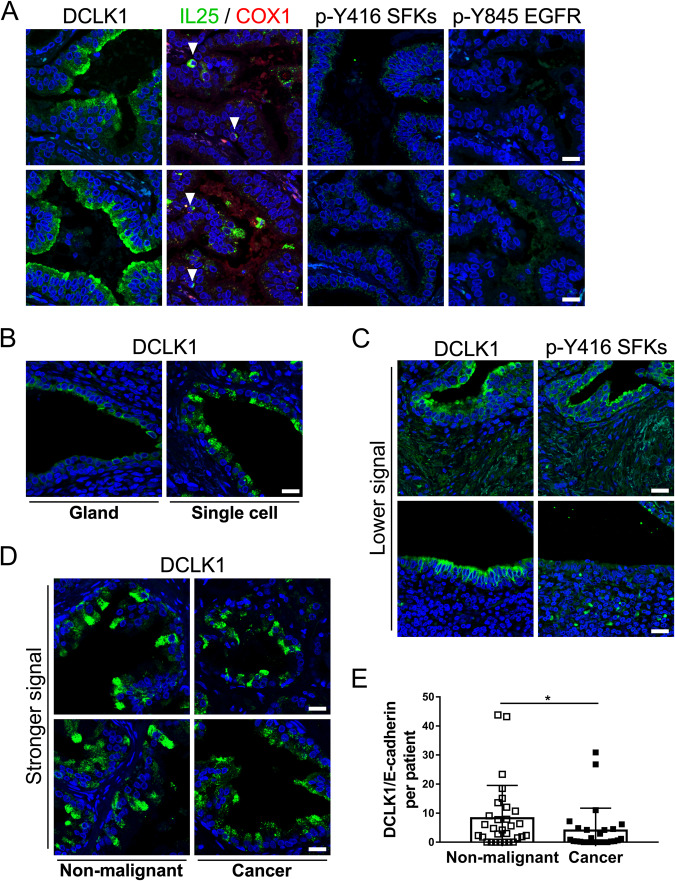
DCLK1 is expressed in localized and diffuse patterns in human prostate cancer, but not in tuft-like cells. **A** DCLK1 does not label human tuft cells. Serial tissue section staining using anti-DCLK1 antibodies revealed a unique staining pattern, not characterized by expression of COX1, or presence of activated EGFR and SFKs. Arrowheads highlight round immune-like cells with high IL-25 staining. Scale bar: 20 μm. **B** DCLK1 expression can be detected throughout glands at lower levels (**C**) or at higher levels in single cells (**D**). Scale bar: 20 μm. **C** Diffuse DCLK1 staining with lower intensity signals. Some of these glands also contain active SFKs, but do not show a tuft-like phenotype. Scale bar: 20 μm. **D** Single cell staining is present both in cancer and adjacent non-malignant tissues. Scale bar: 20 μm. **E** Single cell staining is higher in non-malignant tissues than in cancers. A prostate cancer TMA was stained with anti-DCLK1 and anti-E-cadherin, and the ratio of DCLK1 to E-cadherin was determined in all cores, and averaged per patient. The graph represents data pulled from all patients. N (non-malignant)=30, N (cancer)=27; Error bars shown as ±SD.(*) *p*-value < 0.05.

In concordance with previous reports describing absence of DCLK1 in human tuft cells [5, 6], we do not observe DCLK1 in COX1+ tuft-like cells in human prostate cancer. However, DCLK1 is detectable in different regions of the prostate, having very high staining intensity on the apical borders, with some cells staining stronger than others (Fig. 5A). Comparison of these regions with regions stained with COX1 and IL-25, or with phosphorylated SFKs and EGFR, indicates the absence of a tuft-like phenotype (Fig. 5A). In these regions, we also detected immune cells that stain strongly for IL-25.

The specificity of DCLK1 staining prompted us to further analyze DCLK1 expression in prostate tumors. We performed immunofluorescent staining of a prostate cancer TMA, containing both non-malignant and cancer cores, and observed two distinct expression patterns of DCLK1 staining, including cytoplasmic/membrane staining of whole glands [32, 33], and single cell staining. When DCLK1 is expressed in solitary cells, the staining intensity is stronger than when a more diffuse staining pattern is detected in glands (Fig. 5B). In glands with lower expression, staining of DCLK1 is sometimes found in glands positive for active SFKs, which do not have tuft-like staining (Fig. 5C). DCLK1+ single-cell staining is present in adjacent non-malignant and cancer tissues (Fig. 5D), in cells that do not have expression of active SFKs, supporting the conclusion that these DCLK1+ single cells do not have a tuft cell phenotype. The analysis of the DCLK1+ single cell staining shows they are not cancer specific but are more abundant in non-malignant tissues (Fig. 5E).

### Tuft cell genes are expressed in a distinct cell population in human prostate cancers

**Fig. 6:**
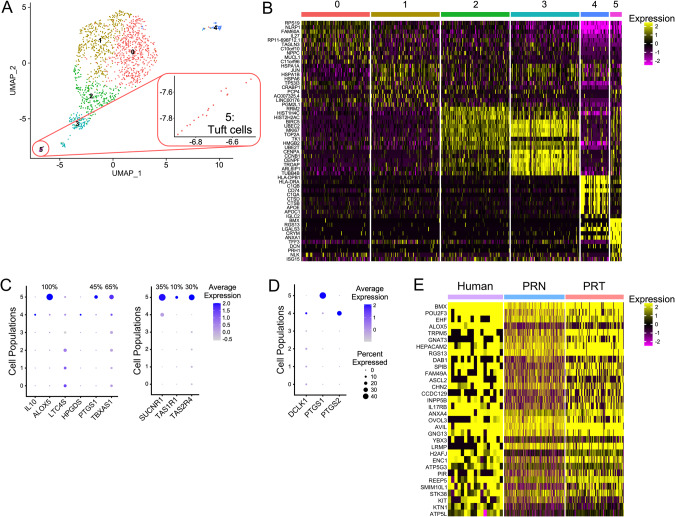
scRNA-seq analysis indicates a tuft cell population in NEPC. **A** A tuft cell population is present in a patient with NEPC. Re-clustering of Dong et al. [34] scRNA-seq data revealed that patient #2 has a cell population, cluster 5, that expresses tuft cell markers. An expanded UMAP of cluster five is shown at the lower right. Different cell types in the human dataset in panels **A**–**D** are represented by: 0-3 – neuroendocrine cells; 4 – immune cells; and 5 – tuft cells. **B** Heatmap of all identified cell populations. Showing up to 100 cells per population, with top 10 genes. **C** Dotplot of genes involved in effector function of tuft cells (left) or sensing receptors (right), with the percentage of the population that expresses them. Human tuft cells do not specifically express *IL10*, *LTC4S* and *HPGDS*, but express *ALOX5*, *PTGS1*, *TBXAS1*. *SUCNR1*, *TAS1R1* and *TAS2R4* are specifically expressed in cluster 5. **D** *PTGS1* (COX1) is a marker of human cancer tuft cells in the prostate. Dotplot shows gene expression of protein markers used for immunohistochemistry to locate tuft cells in Fig. 4. **E** Heatmap of mutual tuft cell genes from two mice and one human tuft cell populations. We identified 32 genes (Table S4) that are significantly expressed in all datasets that can be used to identify prostate tuft cells from mice and men.

Yamada et al. identified expression of tuft cell markers in patients with prostate adenocarcinomas [21]. We examined tuft cell marker expression in available scRNA-seq datasets to identify markers present in human prostate cancer tuft cells. Analysis of datasets from human patients revealed one patient (patient #2) with a neuroendocrine phenotype of CRPC, from Dong et al. (GSE137829 [34]) with clustering of the genes specific for tuft cells. Reclustering of patient #2 scRNA-seq data revealed tuft cell gene expression in population 5 (Fig. 6A, B; Table S4) that exclusively express effector enzymes with roles in synthesis of bioactive lipids, *ALOX5*, *PTGS1* and *TBXAS1* (Fig. 6C). *IL25* and *CHAT* are not detected in this dataset, while *IL10* is not restricted to tuft cells. Among sensing receptors, *SUCNR1*, *TAS1R1* and *TAS2R4* were detected in human prostate tuft cells (Fig. 6C). Human prostate tuft cells specifically express *PTGS1* in comparison to other cell populations, and this confirms that protein expression of this marker (COX1) can be used to detect human tuft cells, together with active kinases (EGFR, SFKs), as shown in Fig. 4. Furthermore, we confirmed that *DCLK1* and *PTGS2* are not markers of human tuft cells (Fig. 6D), as shown in Fig. 4A. Combining mouse and human prostate cancer tuft cell gene expression data, we have identified unique markers for tuft cells in prostate cancer (Fig. 6E; Table S4).

### Tuft cells express unique receptors and ligands that may modulate communication with the tumor microenvironment

**Fig. 7:**
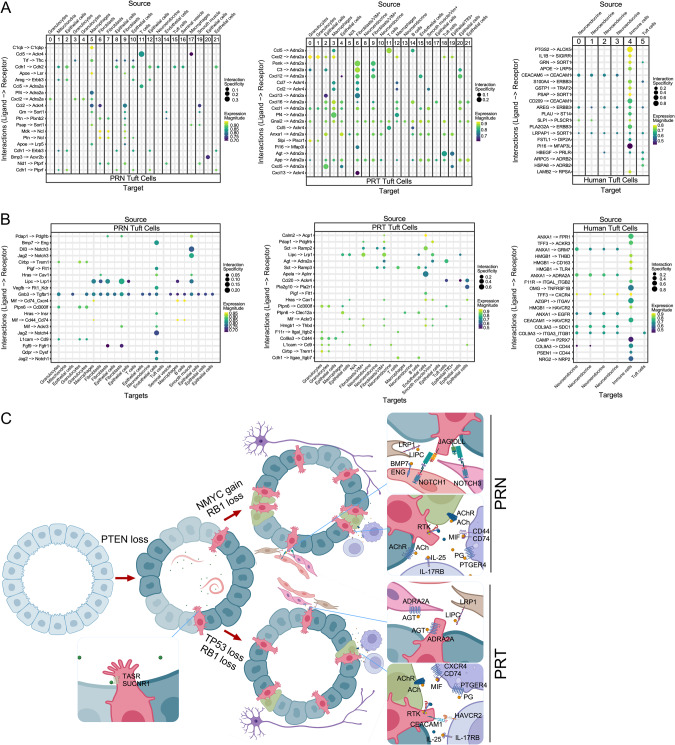
Possible pathways of communication between prostate tuft cells and their environment based on gene expression data. Receptors or ligands that are enriched or specifically expressed in tuft cells were used to map cell-cell interactions in all datasets. **A** Cell-cell interactions between tuft cells and other populations in prostate cancer, highlighting the ligands expressed in all cell types that target receptors expressed in tuft cells. The size of the dot represents the interaction specificity defined by NATMI [69] as uniquely expressed by the interacting pair, while color represents expression magnitude defined by LRscore [70]. Absence of dots in the plots indicate that the interacting genes are expressed in the less than 10% of both cell populations. The top 20 interacting pairs for each dataset show that tuft cell receptors are mostly targeted by ligands expressed by fibroblasts and immune cells. **B** Cell-cell interactions, highlighting the ligands expressed by tuft cells targeting receptors on all cell types in the datasets. Only ligands that are enriched or specifically expressed in tuft cells are used to map interactions. The top 20 interacting pairs show that tuft cell ligands target immune cells in all datasets, but also endothelial and smooth muscle cells in PRN and PRT, and neuroendocrine cells in PRT and human cancer. **C** Expression of sensing receptors on tuft cells suggests their ability to respond to environmental cues. Both PRN and PRT tuft cells express genes that may drive pro-tumorigenic and immunosuppressive signaling. Tuft cells may signal to fibroblasts (Lipc-Lrp1), smooth muscle and endothelial cells (Jag/Dll-Notch, Bmp7-Eng and Agt-Adra2a), immune cells (Ceacam1-Havcr2, Mif-Cd44/Cd74, Mif-Cd74/Cxcr4, Il25-Il17rb and prostaglandin (PG)-Ptger4), and to other epithelial cell types including neuroendocrine cells through ACh signaling to different acetylcholine receptors (AChR). They also receive signals from other cell types through RTKs and Adra2a receptors. Figure 7C was created using BioRender.com.

The availability of computational tools enabled us to further explore unique characteristics of tuft cells in prostate cancer. LIANA (LIgand-receptor ANalysis frAmework), uses the resources and methodologies of different cell-cell communication tools and gives the average ranking of all tools combined [35]. We focused on receptors expressed in tuft cells that recognize ligands coming from all cell populations, or ligands synthesized by tuft cells that target all cell types present in the datasets. Since LIANA does not discriminate between cell-specific gene expression or genes expressed in majority of cell types, we first extracted significant interactions between tuft cells and other populations, and from these data we extracted ligands and receptors that are enriched and specifically expressed in tuft cells (Fig. S2, S3). Subsequently, we used these genes to identify the enriched interaction pairs and specific cell populations with which tuft cells communicate (Fig. 7A, B; Table S5).

We examined signaling interactions between tuft cells and the tumor microenvironment in mouse prostate from PRN [19] and PRT [22] scRNA-seq datasets. Ligands from number of cell types may activate receptors in tuft cells (Fig. 7A). Tuft cells express receptor tyrosine kinases (RTKs) such as *Erbb3*, *Kit* and ephrin receptors, as well as adrenergic receptor subunit *Adra2a* and activin receptors (Fig. 7A, Table S5). Tuft cells that arise in PRN and PRT prostates differ in ephrin receptor expression; they are both enriched for expression of *Epha1*, and PRT is enriched for *Epha4*, while PRN tuft cells specifically express *Epha7* (Fig. S2). They also express receptors and co-receptors of the Wnt pathway, such as *Lrp5*, *Fzd3*, *Fzd7* with *Lrp10* enriched in PRT (Fig. 7A, S2). Furthermore, tuft cells are enriched for *Ackr4* (Atypical Chemokine receptor 4), *Tfrc* (Transferrin Receptor), *Sort1* (Sortilin-1). Additional genes encoding receptors specifically expressed in each tuft cell population are highlighted in Fig. S2. The majority of ligands targeting these receptors on tuft cells are expressed in fibroblasts, macrophages and some epithelial populations (Fig. 7A, S4A, S4B).

Using LIANA, we identified ligands expressed by tuft cells that target other cell populations in PRN and PRT cancer models (Fig. 7B). We identified novel ligands expressed in tuft cells, such as: *Lipc* (Lipase C, hepatic), *Mif* (Macrophage migration inhibitory factor), *Vegfb* (Vascular endothelial growth factor B) and *Fgf9* (Fibroblast growth factor 9). *Fgf9* from tuft cells is predicted to target *Fgfr1* on fibroblasts in both PRN and PRT, and epithelial cells through *Fgfr1* or *Fgfr2* in PRT cancer model (Fig. 7B, Table S5). We also detect *Ptpn6* and *L1cam* as PRT tuft cell specific and PRN enriched ligands, genes that were identified as tuft cell markers [25, 36]. Furthermore, PRN tuft cells specifically express *Jag2* and *Dll3*, that are proposed to interact with *Notch1,3-4* expressed in endothelial and smooth muscle cells (Fig. 7B). In addition to these ligands, in Fig. S3 we highlighted additional ligands specific to each tuft cell population, such as *Frem1* and *Fras1* (PRN specific), *Agt* (PRT specific).

We explored human tuft cell interactions and found they share similarities with both PRN and PRT tuft cells and communicate with immune cells (Fig. 7A and B, right panels; Fig. S4C). Like PRN and PRT mouse tuft cells, human tuft cells express ERBB and WNT pathway proteins such as *ERBB3*, *FZD3*, *LRP5*, and *LRP10* (Fig. S2C, Table S5). Human tuft cells express some ligands that are expressed in either PRN or PRT tuft cells (Fig. S3). Like mouse PRT prostates, human prostate tuft cells also express ligands on their surface such as *CEACAM1* (Carcinoembryonic antigen cell adhesion molecule 1), and gene coding collagen *COL9A3* (Collagen type IX alpha 3 chain), while they specifically express *ANXA1*, *TFF3*, *OMG*, and other genes highlighted in Fig. S3C.

The expression of receptors for non-peptide tuft cell ligands, such as acetylcholine, prostaglandin and leukotriene receptors, suggest additional ways that tuft cells may modulate the microenvironment in advanced prostate cancer. Receptors for acetylcholine are primarily found on neuroendocrine cells in all scRNA-seq datasets (Fig. S5), with addition of several epithelial populations in PRN model (Fig. S5A). Receptors for eicosanoids in PRN and PRT cancer models (Fig. S5A, B) are primarily located in immune cells and fibroblasts: *Fpr2* is located in granulocytes, *Cysltr1* and *Ptger4* in macrophages (and B cells in PRT), *Ptger3* in fibroblasts, while *Ptgir* is found in smooth muscle cells. *CYSLTR1* and *PTGER4* are primarily expressed by immune cells in human cancers, while *LTB4R*, *PTGER1* and *PTGER3* are found in neuroendocrine and tuft cells (Fig S5C). Together these data underscore the important roles that tuft cells may have in regulating the prostate tumor microenvironment. Proposed interactions that need to be experimentally validated are schematically summarized in Fig. 7C.

## Discussion

Tuft cells with both tumor suppressive and/or promoting functions have been described in cancers [1]. We identified tuft cell populations that are upregulated in prostate cancer using immunohistochemistry (Figs. 1 and 4) and bioinformatic analyses (Figs. 2, 3 and 6). In the scRNA-seq datasets from the PRN and PRT mouse models, we detected distinct cell populations that express *Pou2f3* and other tuft cell markers (Fig. 2). We show that tuft cell-related marker gene expression increases with age and cancer progression in PRN mice (Fig. 3). This was not observed in PRT mice, perhaps due to limitations of RNA-seq to detect subtle changes in gene expression in a small subpopulation of cells, or the limited number of aging mice used, or other possible differences in tuft cell expansion in this model. Chan et al. show that the *Pou2f3*+ population in PRT mice increases from 8- and 9-week old to 12-week old mice, but the size of this population is reduced again at 16 weeks [22]. Sawyers and colleagues have indicated they will be analyzing the *Pou2f3* population in their dataset in more depth, which may give better insight into PRT tuft cells [22].

Tuft cells function as a surveillance system for extracellular pathogens such as helminths and protists. Through G protein coupled receptors expressed on their surface, tuft cells detect changes in their environment, and activate a type 2 immune response. Prostate cancer has been associated with inflammation and the presence of microorganisms within the tissue [37–40]. *Schistosoma haematobium*, a helminth [38, 39, 41–43] and *Trichomonas vaginalis*, a protist [40, 44] are the most common pathogens that infect the prostate. While a causal correlation between prostate cancer and infection has not been clearly demonstrated [38, 44], there have been reports of early-onset disease in patients with infection with these microorganisms [41–43]. We did not detect tuft cells in healthy prostates (Fig. 1), and it will be interesting to determine if they are upregulated upon prostate infection. However, we show that neoplastic changes lead to an increase in tuft cells in the prostate, similar to findings in other cancers [1]. Receptors on the surface of prostate cancer tuft cells can detect environmental changes (Figs. 2F and 6D), which may include factors secreted from cancer cells, the cancer microenvironment, or by pathogens to promote tumorigenesis.

Our bioinformatic analyses identified several ligands expressed in tuft cells, which may target immune cells to create an inflammatory immunosuppressive microenvironment (Figs. 2, 6 and 7B). Tuft cells in mouse models of prostate cancer express *Il25*, and *Il10* that may have immunosuppressive roles, as well as enzymes for eicosanoid synthesis for production of other immunomodulatory molecules. Prostaglandin signaling through *PTGER4* (Fig. S5) could promote angiogenesis and infiltration of immune cells and create an immunosuppressive environment [45]. The novel tuft cell ligands encoded by *Frem1*, *Fras1* and *Agt* may have tumor suppressor functions, but have also been found to correlate with immune infiltration and metastasis [46–49], while *Mif* could suppress anti-tumor immunity of infiltrating immune cells [50] (Fig. 7B). An interesting ligand expressed in cancer tuft cells is *CEACAM1* which can modulate and inhibit responses of several immune cell types such as Natural Killer (NK) or T cells [51, 52]. *CEACAM1* can interact with various receptors, and we identified one possible interaction between tuft cell and immune cells, *CEACAM1*-*HAVCR2* in PRT and human cancers (Fig. 7B, Table S5). In the PRT mouse model, *Havcr2* (T cell marker) is expressed in populations expressing markers for B cells (cluster 14) and macrophages (cluster 3) (Table S5), but we cannot exclude that these populations are heterogeneous, having some other smaller populations of T cells.

We determined that prostate tuft cells appear around nerve fibers in human cancers (Fig. 4), which suggests crosstalk between them. The importance of neural invasion in cancer has been gaining attention in recent years, particularly in prostate cancer [53, 54]. Both sympathetic adrenergic and parasympathetic cholinergic fibers have been shown to promote early prostate tumorigenesis or invasion and metastasis, respectively [55]. Tuft cells may contribute to both cholinergic and adrenergic signaling in advanced prostate cancer. We found that tuft cells express *Chat* for synthesis of acetylcholine (Fig. 2E), while other cell populations in tumors, particularly neuroendocrine cells and other epithelial cells, express cholinergic receptors (Fig. S5). Tuft cells also express adrenergic receptors and/or their ligands (Fig. 7A, B; S2, S3), suggesting autocrine signaling and adrenergic communication with other cell populations.

Tuft cells appear to be signaling hubs, and we detected activation of SFKs and EGFR specifically in tuft cells (Figs. 1 and 4), as well as expression of additional intracellular and receptor kinases (Figs. 2D, 6F; Table S5). Interestingly, tuft cells may share characteristics of cancer stem-like cells (Fig. 6F, S2), cells that have been identified based on high c-Kit expression and have increased migratory and invasion potential [56]. Furthermore, through expression of ligands for Notch and Bmp7 (Fig. 7B), tuft cells could target Notch1/3 and Endoglin (*Eng*) on endothelial and smooth muscle cells to promote cancer progression through promotion of angiogenesis [57, 58]. We also detected expression of other TGF-beta family members such as *Acvr1b* and *Acvr2b*, that may promote metastasis and the EMT [59].

In prostate cancer, we find that increased numbers of tuft cells may indicate more aggressive disease. In addition, upregulation of tuft cells resulting from infection or neoplastic transformation may further promote cancer progression (Fig. 7C). Additional studies are needed to determine if tuft cell markers may serve as prognostic indicators that reveal new therapeutic vulnerabilities for targeting this common disease.

## Materials and methods

### Mice

All animal experiments were approved by the University of Illinois at Chicago Institutional Animal Care and Use Committee. All mice were maintained under specific pathogen-free conditions. Generation of *PB-Cre4;Pten*^*fl/fl*^ (B6.Cg-Tg(*Pbsn-cre)4Prb*;*Pten*^loxP/loxP^) mice has been described [60, 61]. Age-matched littermates with floxed *Pten*, either expressing *PB-Cre4* or controls, were sacrificed at 4 and 8 months. Paraffin-embedded whole prostate tissues were stained for tuft cell-related protein expression. The number of tuft cells is quantified as the number of DCLK1+ cells per area. For 4- and 8-month old mice, 8 and 15 random areas of anterior prostate were analyzed, respectively.

### Patient samples

Human tissues used for analysis include a human prostate cancer tissue microarray (TMA) developed by Dr. Larisa Nonn (University of Illinois at Chicago) [62] and CRPC samples isolated by transurethral resection of the prostate (TURP) [63]. Human tissue use was approved by The University of Illinois at Chicago Institutional Review Board. The TMA consists of 102 biopsy cores, obtained from 20 African American and 11 European American patients. Hematoxylin and eosin (H&E) stained cores were analyzed and divided into cancer or non-malignant group by a pathologist. ImageJ was used for the quantification of fluorescence signal for DCLK1 and E-cadherin. Quantity is expressed as the fluorescence of DCLK1 per area of epithelium (E-cadherin positive) for each core.

### Immunofluorescence

Tissue staining was performed as described by Alwanian and colleagues [63]. Antibodies and reagents used are shown in Table S1. Images were taken with a Zeiss LSM700 Confocal microscope. Images for quantification were taken with Leica DM8 fluorescent microscope at ×10 magnification.

### Data analysis and statistics

Publicly available scRNA-seq and RNA-seq data were obtained from GEO (Gene Expression Omnibus, https://www.ncbi.nlm.nih.gov/geo/) or directly from authors (Brady et al. [19] and Dardenne et al [18]), and include the following datasets: GSE158467 and GSE158468 [19], GSE210358 [22], GSE86532 [18], GSE137829 [34], GSE90891 [31]. Data were analyzed using R software. For the analysis of scRNA-seq data, we used the Seurat package [64], and for interaction analysis we used LIANA [35]. Cell populations were determined using annotations from published research [19, 22, 65]. For RNA-seq analysis we used ComBat-seq [66] for batch effect correction of raw counts, and edgeR [67] and ComplexHeatmap packages [68] for further analysis and visualization. False Discovery Rate (FDR) was used to determine significant changes in gene expression between mice groups. Statistical analyses for quantification data were performed using GraphPad Prism software version 7 (La Jolla, CA); two-way ANOVA and Mann-Whitney tests were used to determine significant differences between groups.

## Supplementary information

Text_Supplemental Materials

Figure S1

Figure S2

Figure S3

Figure S4

Figure S5

Table S1

Table S2

Table S3

Table S4

Table S5

## Data Availability

The datasets used in this study are listed in sections “Material and Methods” and “Supplemental Material and Methods.” Sharing of our data is not applicable to this article as no datasets were generated.
