# Supplementary material for: Appearance of tuft cells during prostate cancer progression
Source: Oncogene. 2023 Jun 29;42(31):2374–85. doi: 10.1038/s41388-023-02743-1 (PMC10374444; doi:10.1038/s41388-023-02743-1)
Supplement: Supplementary file 1 — Text_Supplemental Materials [file 41388_2023_2743_MOESM1_ESM.docx]

**SUPPLEMENTARY INFORMATION FOR:**

**Appearance of tuft cells during prostate cancer progression**

Katarina Vlajic^1^, Hannah Pennington Kluger^1^, Wenjun Bie^1^, Bradley J. Merrill^1,2^, Larisa Nonn^2,3^, Andre Kajdacsy-Balla^2,3^, and Angela L. Tyner^1,2^

1. **Supplementary Materials and methods.**
2. **Supplementary figure legends.**
3. **Supplementary table legends.**
4. **Supplementary references.**
5. **Supplementary figures S1-S5.**
6. **Supplementary tables S1-S5.**

**SUPPLEMENTARY MATERIALS AND METHODS**

**Data Analysis**

Additional publicly available scRNA-seq datasets used for screening of tuft cells were obtained from GEO website (<https://www.ncbi.nlm.nih.gov/geo/>), including: GSE176031 [1], GSE193337 [2], GSE205765 [3], GSE143791 [4], GSE168669 [5], GSE157703 [6], GSE141445 [7], GSE188318, GSE130318 [8], GSE117403 [9].

To explore differences in tuft cell populations, we compared Tp53 target genes with PRN and PRT tuft cell gene expression (Table S2). The exhaustive list of Tp53 target genes is from Fischer, using threshold Sum direct regulation score >2 or <-2 [10]. For NMYC regulated genes, we used genes identified by Valentijn and colleagues [11]. To explore tuft cell types, we compared PRN and PRT specific genes with type-1 and type-2 tuft cell markers identified in small intestine and lungs [12, 13].

To explore differences between PRN and PRT tuft cells and find mutual markers, we compared all significantly expressed genes in tuft cell clusters from scRNA-seq data [14, 15] (Table S2). Mutually expressed genes with adjusted p-values < 10^-100^ from both datasets (Table S2) were compared to find common tuft cell markers. For the analysis of tuft cell marker gene upregulation with disease severity in RNA-seq datasets, we used more strict selection, with adjusted p-values < 10^-200^. The data shown in tables (Table S3) is presented as XvsY (ie X = WT, Y = P); positive logFC indicate higher expression in X, negative logFC indicate higher expression in Y.

LIANA was used to examine interactions between cell populations [16]. The threshold for source and target genes from tuft cell populations was set at RRA < 0.05. Unique source (ligands) and target (receptors) genes were extracted for each tuft cell population. For each of the gene sets, the avg. exp. scaled > 0.5 was used to extract genes enriched in tuft cells, and the avg. exp. scaled > 2 was used to extract genes specific for tuft cells.

**SUPPLEMENTARY FIGURE LEGENDS**

**Figure S1: Characterization of populations from PRN and PRT mice.** A) Heatmap of all populations obtained upon re-clustering of Brady et al. [15] scRNA-seq data. Tuft cell genes are clustering in population 15. B) Heatmap of all populations obtained upon re-clustering of Chan et al. [14] scRNA-seq data. Tuft cell genes are clustering in population 18. C) Expression of protein markers that are used to identify the tuft cell population in Fig. 1 in each of the dataset. Overlap of these three markers can be used to locate tuft cells in the tissues.

**Figure S2:** **Receptors expressed in tuft cells, used for exploration of cell-cell interactions.** Dotplots of receptors expressed in tuft cells that build cell-cell interactions with other cell types in all datasets. Receptors were identified using LIANA, and then selected for ones enriched or specifically expressed in tuft cells. Included receptors were enriched in all three used datasets, enriched in two out of three datasets, or specifically expressed in either of three. Tuft cell populations are boxed in red. Some genes could be enriched or specifically expressed in some of the populations, but no interacting partners were found in that dataset (e.g., *IL17RB* in human tuft cells). A) PRN cancer model [15], B) PRT cancer model [14], C) Human cancer [17].

**Figure S3: Ligands expressed in tuft cells, used for exploration of cell-cell interactions.** Dotplots of ligands expressed in tuft cells that target receptors expressed in other cell types in all datasets. Ligands were identified from interaction pairs using LIANA, and then selected for the ones that are enriched or specifically expressed in tuft cells. Shown are ligands that were enriched in all three used datasets, enriched in two out of three datasets, or specifically expressed in either of three. Tuft cell populations are boxed in red. A) PRN cancer model [15], B) PRT cancer model [14], C) Human cancer [17].

**Figure S4: Overview of significant interactions detected using LIANA.** A chord diagram was used to visualize ligands coming from tuft cells targeting all cell types in datasets; or ligands coming from all cell types targeting receptors on tuft cells. Only significant interaction pairs, with enriched or specifically expressed genes in tuft cells were used to generate the chord diagram. All cell populations identified in Fig. 2 and Fig. 6 are labeled in the diagram. “Tuft L” represents the percentage of ligands coming from tuft cells targeting that specific cell population; “Tuft R” represents the percentage of receptors expressed in tuft cells that are interacting with ligands coming from that specific population. Percentages are shown only for cell populations that interact most frequently with tuft cells. A) Chord diagram of interactions present in PRN cancer model [15]; B) Chord diagram of interactions present in PRT cancer model [14]; C) Chord diagram of interactions present in human cancer [17].

**Figure S5: Expression of receptors for acetylcholine and eicosanoids in all datasets.** Dotplots showing receptors for acetylcholine and eicosanoids in all datasets. A) PRN cancer model [15], B) PRT cancer model [14], C) Human cancer [17].

**SUPPLEMENTARY TABLE LEGENDS**

**Table S1.** List of antibodies and reagents used for immunofluorescence staining.

**Table S2.** Analysis of Brady et al. [15] and Chan et al. [14] scRNA-seq data from PRN and PRT mice. Data are related to Figs. 2 and S1, and Table 1.

**Table S3.** Analysis of tuft cell marker gene expression in studies GSE86532 [18], GSE90891 [19] and GSE158467 [15] after adjustment for batch effect. Data are related to Fig. 3.

**Table S4.** Analysis of Dong et al. [17] scRNA-seq data from patient number 2. Data are related to Fig. 6.

**Table S5.** Analysis of cell-cell interaction between tuft cells and other populations in all scRNA-seq datasets [14, 15, 17] using LIANA. Data are related to Figs. 7 and S4.

**REFERENCES**

1 Song H, Weinstein HNW, Allegakoen P, Wadsworth MH, Xie J, Yang H *et al*. Single-cell analysis of human primary prostate cancer reveals the heterogeneity of tumor-associated epithelial cell states. *Nature Communications* 2022; 13: 141.

2 Heidegger I, Fotakis G, Offermann A, Goveia J, Daum S, Salcher S *et al*. Comprehensive characterization of the prostate tumor microenvironment identifies CXCR4/CXCL12 crosstalk as a novel antiangiogenic therapeutic target in prostate cancer. *Mol Cancer* 2022; 21: 132.

3 Sandhu HS, Portman KL, Zhou X, Zhao J, Rialdi A, Sfakianos JP *et al*. Dynamic plasticity of prostate cancer intermediate cells during androgen receptor-targeted therapy. *Cell Rep* 2022; 40: 111123.

4 Kfoury Y, Baryawno N, Severe N, Mei S, Gustafsson K, Hirz T *et al*. Human prostate cancer bone metastases have an actionable immunosuppressive microenvironment. *Cancer Cell* 2021; 39: 1464-1478.e1468.

5 Taavitsainen S, Engedal N, Cao S, Handle F, Erickson A, Prekovic S *et al*. Single-cell ATAC and RNA sequencing reveal pre-existing and persistent cells associated with prostate cancer relapse. *Nat Commun* 2021; 12: 5307.

6 Ma X, Guo J, Liu K, Chen L, Liu D, Dong S *et al*. Identification of a distinct luminal subgroup diagnosing and stratifying early stage prostate cancer by tissue-based single-cell RNA sequencing. *Mol Cancer* 2020; 19: 147.

7 Chen S, Zhu G, Yang Y, Wang F, Xiao YT, Zhang N *et al*. Single-cell analysis reveals transcriptomic remodellings in distinct cell types that contribute to human prostate cancer progression. *Nat Cell Biol* 2021; 23: 87-98.

8 McCray T, Moline D, Baumann B, Vander Griend DJ, Nonn L. Single-cell RNA-Seq analysis identifies a putative epithelial stem cell population in human primary prostate cells in monolayer and organoid culture conditions. *Am J Clin Exp Urol* 2019; 7: 123-138.

9 Henry GH, Malewska A, Joseph DB, Malladi VS, Lee J, Torrealba J *et al*. A Cellular Anatomy of the Normal Adult Human Prostate and Prostatic Urethra. *Cell reports* 2018; 25: 3530-3542.e3535.

10 Fischer M. Census and evaluation of p53 target genes. *Oncogene* 2017; 36: 3943-3956.

11 Valentijn LJ, Koster J, Haneveld F, Aissa RA, van Sluis P, Broekmans MEC *et al*. Functional MYCN signature predicts outcome of neuroblastoma irrespective of <i>MYCN</i> amplification. *Proceedings of the National Academy of Sciences* 2012; 109: 19190-19195.

12 Haber AL, Biton M, Rogel N, Herbst RH, Shekhar K, Smillie C *et al*. A single-cell survey of the small intestinal epithelium. *Nature* 2017; 551: 333-339.

13 Montoro DT, Haber AL, Biton M, Vinarsky V, Lin B, Birket SE *et al*. A revised airway epithelial hierarchy includes CFTR-expressing ionocytes. *Nature* 2018; 560: 319-324.

14 Chan JM, Zaidi S, Love JR, Zhao JL, Setty M, Wadosky KM *et al*. Lineage plasticity in prostate cancer depends on JAK/STAT inflammatory signaling. *Science* 2022; 377: 1180-1191.

15 Brady NJ, Bagadion AM, Singh R, Conteduca V, Van Emmenis L, Arceci E *et al*. Temporal evolution of cellular heterogeneity during the progression to advanced AR-negative prostate cancer. *Nature Communications* 2021; 12: 3372.

16 Dimitrov D, Türei D, Garrido-Rodriguez M, Burmedi PL, Nagai JS, Boys C *et al*. Comparison of methods and resources for cell-cell communication inference from single-cell RNA-Seq data. *Nature Communications* 2022; 13: 3224.

17 Dong B, Miao J, Wang Y, Luo W, Ji Z, Lai H *et al*. Single-cell analysis supports a luminal-neuroendocrine transdifferentiation in human prostate cancer. *Commun Biol* 2020; 3: 778.

18 Dardenne E, Beltran H, Benelli M, Gayvert K, Berger A, Puca L *et al*. N-Myc Induces an EZH2-Mediated Transcriptional Program Driving Neuroendocrine Prostate Cancer. *Cancer cell* 2016; 30: 563-577.

19 Ku SY, Rosario S, Wang Y, Mu P, Seshadri M, Goodrich ZW *et al*. Rb1 and Trp53 cooperate to suppress prostate cancer lineage plasticity, metastasis, and antiandrogen resistance. *Science* 2017; 355: 78-83.
