# Supplementary figures and images for: Appearance of tuft cells during prostate cancer progression
Source: Oncogene. 2023 Jun 29;42(31):2374–85. doi: 10.1038/s41388-023-02743-1 (PMC10374444; doi:10.1038/s41388-023-02743-1)

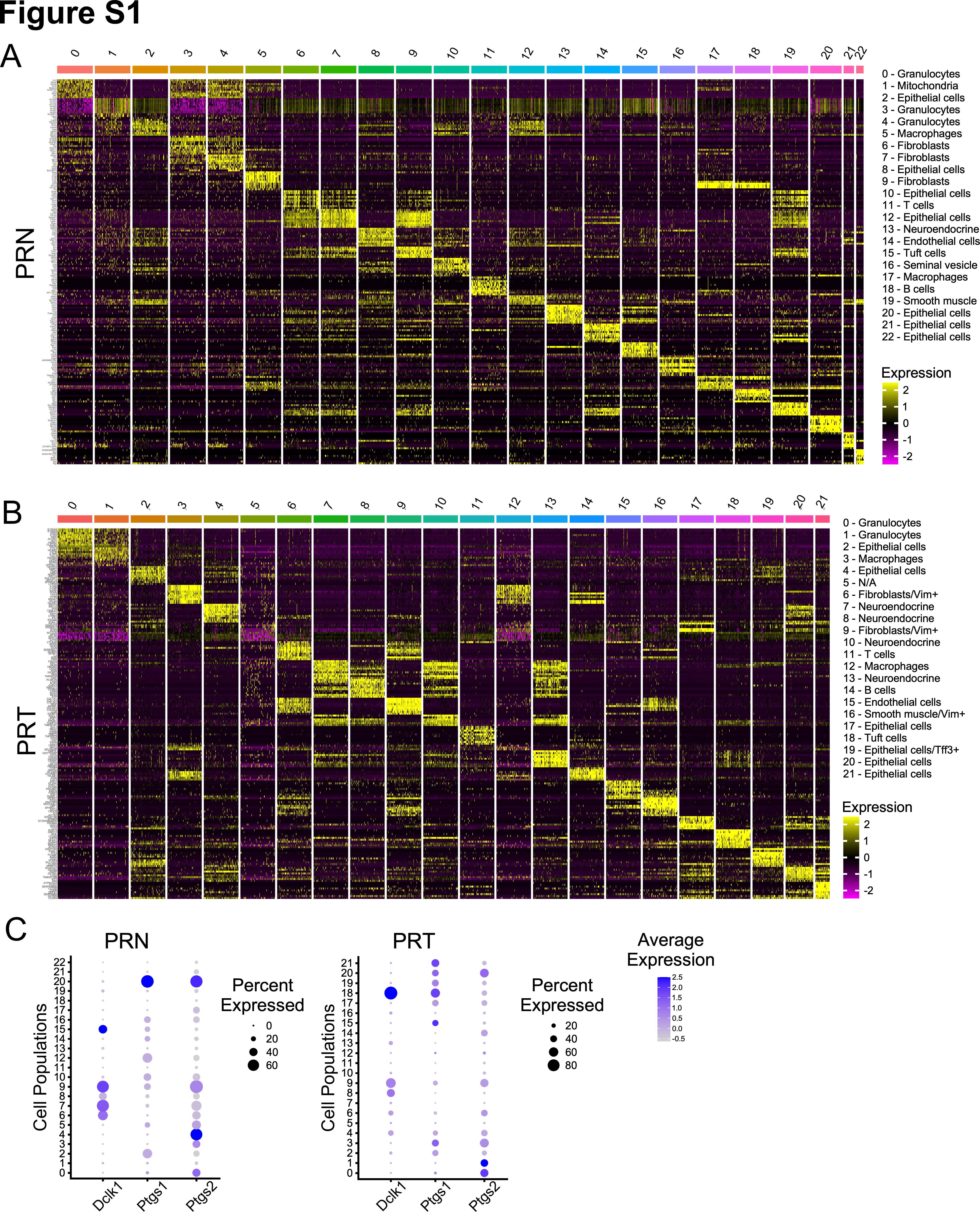

Supplement: Supplementary file 2 — Figure S1 [file 41388_2023_2743_MOESM2_ESM.tif]

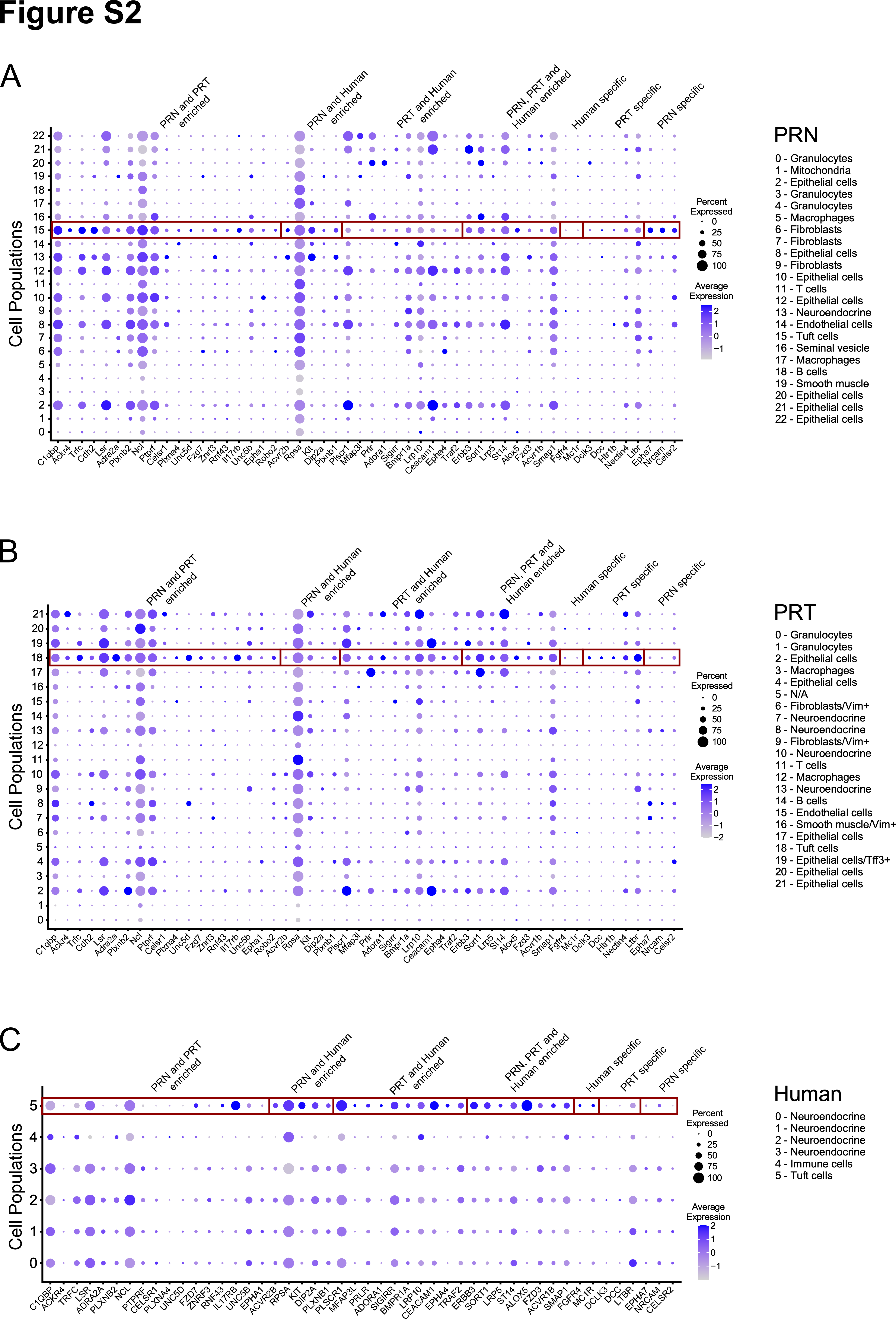

Supplement: Supplementary file 3 — Figure S2 [file 41388_2023_2743_MOESM3_ESM.tif]

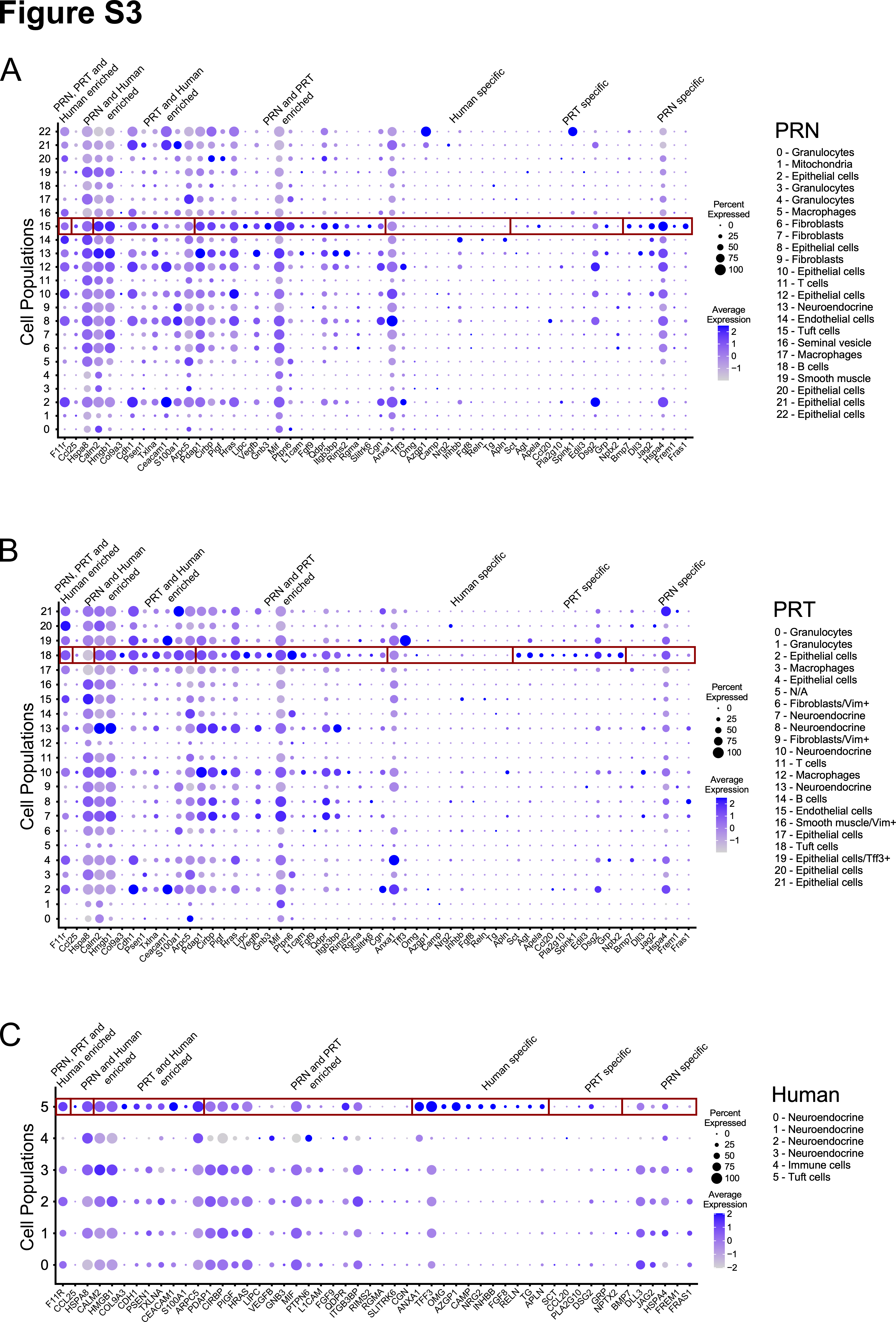

Supplement: Supplementary file 4 — Figure S3 [file 41388_2023_2743_MOESM4_ESM.tif]

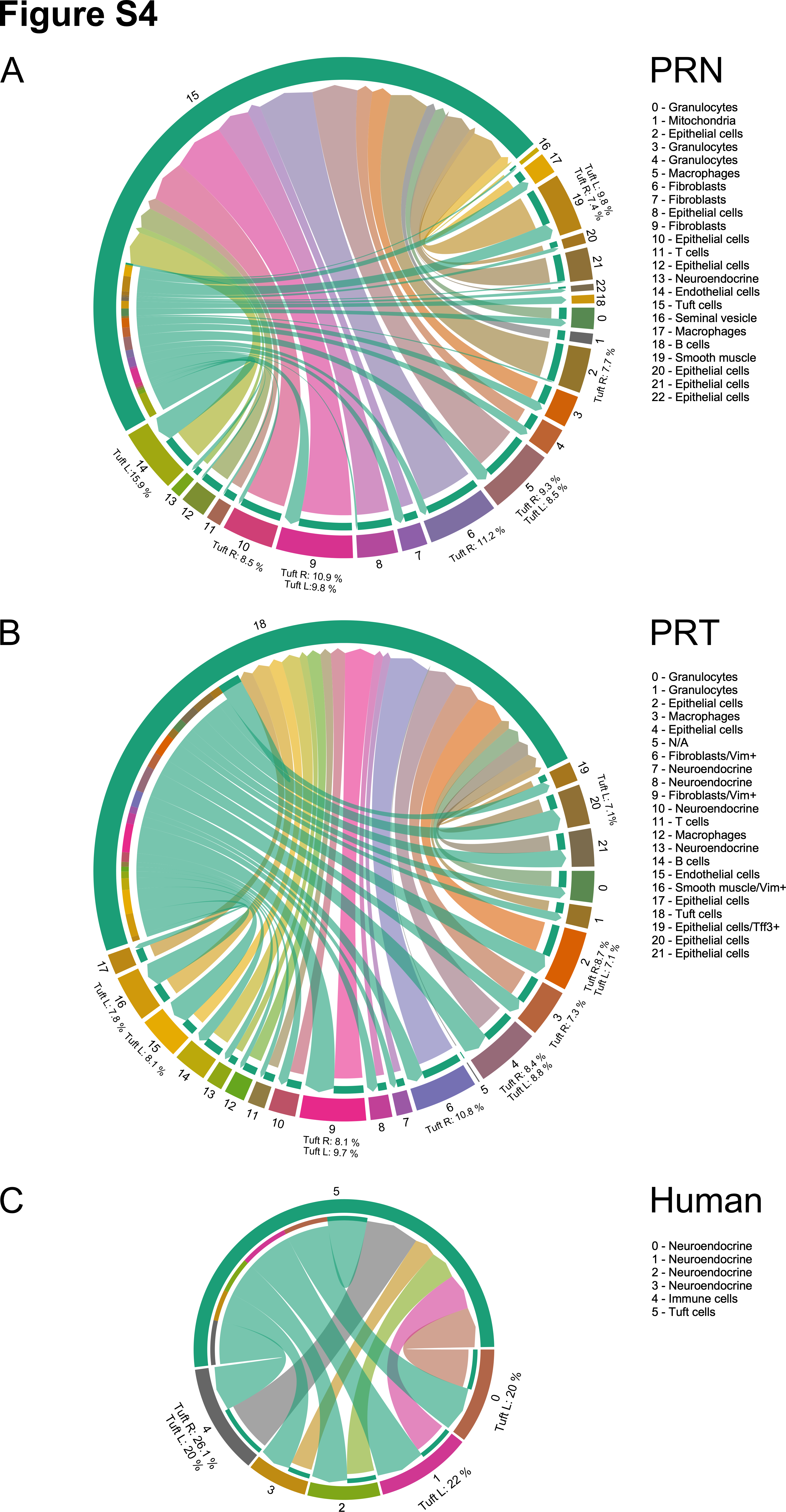

Supplement: Supplementary file 5 — Figure S4 [file 41388_2023_2743_MOESM5_ESM.tif]

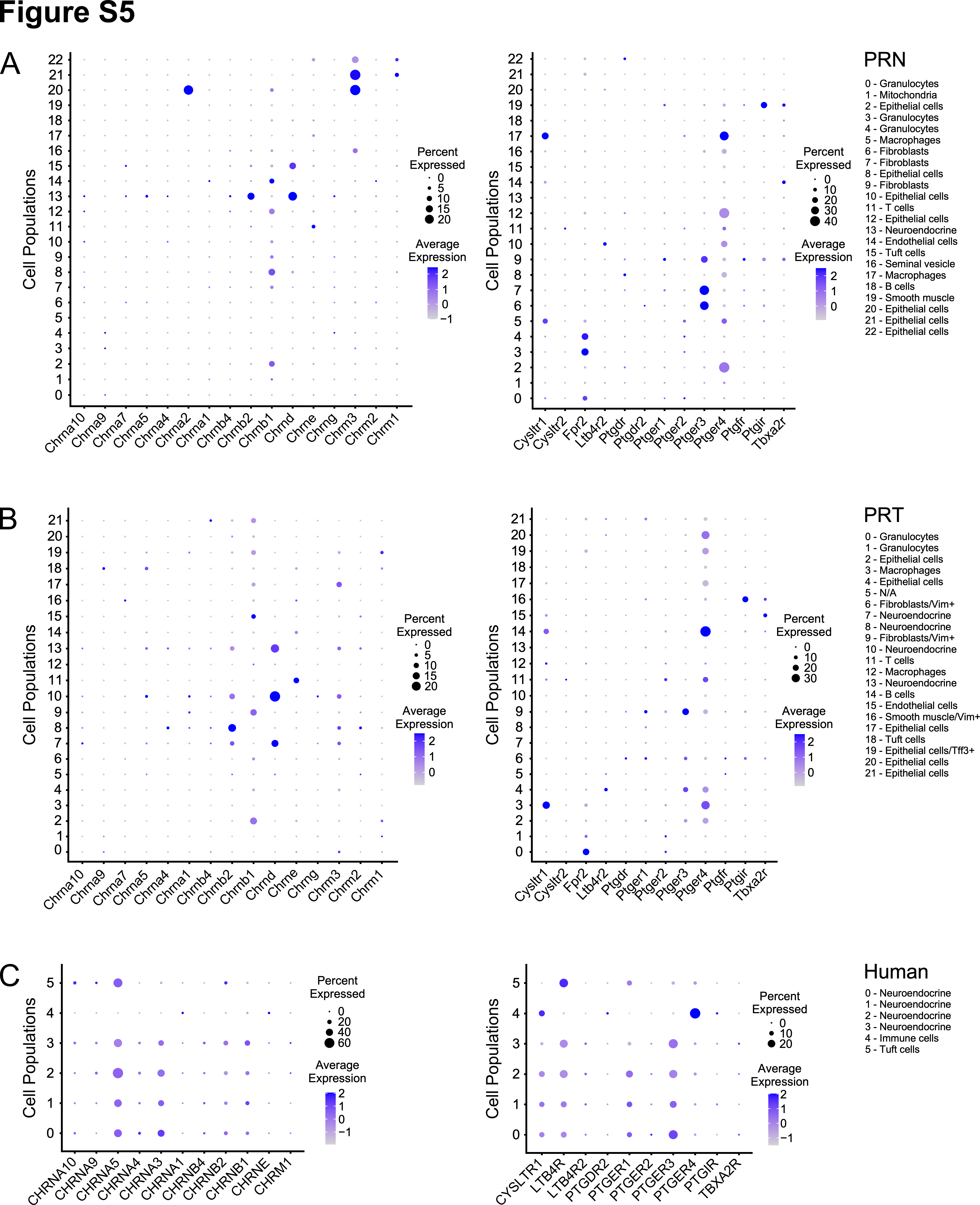

Supplement: Supplementary file 6 — Figure S5 [file 41388_2023_2743_MOESM6_ESM.tif]
