# Supplementary material for: Appearance of tuft cells during prostate cancer progression
Source: Oncogene. 2023 Jun 29;42(31):2374–85. doi: 10.1038/s41388-023-02743-1 (PMC10374444; doi:10.1038/s41388-023-02743-1)
Supplement: Supplementary file 7 — Table S1 [file 41388_2023_2743_MOESM7_ESM.docx]

| **Antibody** | **Dilution** | **Catalogue number** | **Company** | **Company location** |
| --- | --- | --- | --- | --- |
| anti-DCLK1 | 1:500 | ab31704 | Abcam | Cambridge, UK |
| anti-p-Y845 EGFR | 1:100 | 2231 | Cell Signaling Technology | Danvers, MA, USA |
| anti-p-Y416 SRC | 1:100 | 2101 | Cell Signaling Technology | Danvers, MA, USA |
| anti-COX1 | 1:100 | 4841 | Cell Signaling Technology | Danvers, MA, USA |
| anti-COX2 | 1:100 | 5153 | Cell Signaling Technology | Danvers, MA, USA |
| anti-IL-25 | 1:100 | MAB1258-SP | R&D Systems | Minneapolis, MN, USA |
| anti-E-Cadherin | 1:200 | sc-7870 | Santa Cruz Biotechnology Inc | Dallas, TX, USA |
| anti-rabit IgG (goat) | 1:200 | BA-1000 | Vector Laboratories | Burlingame, CA, USA |
| anti-mouse IgG (horse) | 1:200 | BA-2000 | Vector Laboratories | Burlingame, CA, USA |
| anti-rabbit IgG Alexa Fluor-594 | 1:100 | 8889 | Cell Signaling Technology | Danvers, MA, USA |
| AffiniPure Fab Fragment Goat Anti-rabbit IgG | 1:30 | 111-007-003 | Jackson ImmunoResearch Laboratories Inc | West Grove, PA, USA |
| streptavidin-Alexa Fluor-488 | 1:500 | S32354 | Invitrogen | Waltham, MA, USA |
| DAPI | 1:5000 | D1306 | Invitrogen | Waltham, MA, USA |
| Vectashield | na | H-1000 | Vector Laboratories | Burlingame, CA, USA |
